# Creating and Delivering Service Value in Medical Imaging Departments, the Marketing Imperative

**DOI:** 10.5334/jbsr.2552

**Published:** 2021-09-21

**Authors:** Bart Claikens

**Affiliations:** 1 AZ Damiaan Oostende, BE

**Keywords:** radiology, hospital, management, marketing

## Abstract

In an era of increased pressure on reducing expenditure in health care and a rising demand for high-quality, safe, accessible, and payable medicine, one must use more valued models. Marketing is a broad approach to build exchange relationships, including all aspects of the organization’s interfaces.

This opinion article takes a first step in revealing the marketability of imaging departments.

## Introduction

**Figure 1:**
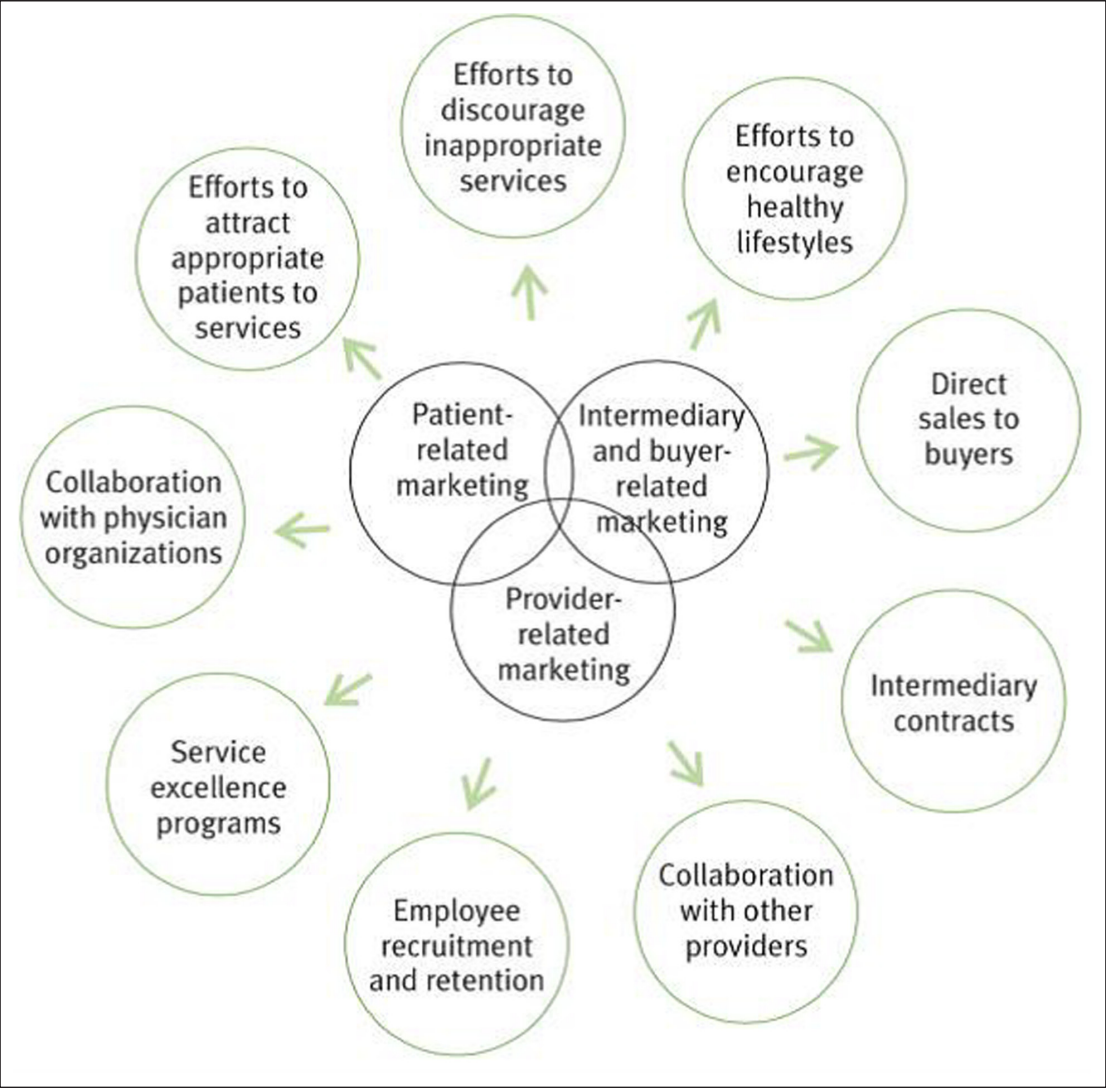
Major marketing directions in hospital organizations.

Imaging departments should ensure an excellent patient experience, with the highest quality. A dedicated marketing strategy is important. Several major hospital marketing directions are known [1] (***Figure 1***).

Implementing marketing is key to staying competitive and keeping up with healthcare regulations. Imaging departments must start thinking beyond their tangible products, like providing images and reports. They should focus on attracting appropriate patients to their services and stand out in medical collaboration.

The hospital industry may have once been considered a slow adopter to the world of inbound marketing, but it’s safe to say that examples of true marketing brilliance are busy making up for lost time [2].

## Creating and Delivering Value

A first step is to identify the marketable service lines. Markets are segmented in subgroups with similar needs, to understand the perspectives of patients and referring physicians, with both qualitative and quantitative approaches.

Differentiations are based on different imaging service modalities. The service lines should be clearly defined.

Diagnosis codes (ICD) are used to group patients based on their health status [3]. A dedicated competitive campaign and an analysis of the access barriers (product/service, price, place, promotion) is set up for each patient group.

Accessible groups with similar preferences are identified, based on their characteristics and expected services required, big enough to matter and small enough to be cohesive.

Mapping the geographic service area for each of the service lines is mandatory. Some of the service lines can only effectively reach individuals within a short distance, and others, much larger areas.

Marketing efforts should be targeted to these service lines, patient groups and geographical spots in a strategy for future patient accessibility. The competitive advantage and the market attractiveness will lead to potential targets.

## Customer Pain Point Analysis

Each imaging modality has distinct needs associated with it, that might create pains for patients or referring physicians.

Long waiting times for patients are not uncommon in many service organizations. Queues are ubiquitous and undesirable. Delay in receiving needed services can cause prolonged discomfort and economic loss for patients and worsening the overall medical conditions, subsequent increasing treatment costs and poor health outcomes. Waiting times are the most frequent mentioned drawbacks and are a unique component related to customer experiences.

Imaging departments must tackle this by managing operational excellence by process design, improving the customer overall experience. Valued key performance indicators will monitor and guide the overall process.

## Value Propositions

Safety is the most crucial value proposition. High quality is the cornerstone of evidence-based and value-based medicine, whereas care services should be always accessible and payable. These value propositions should be embedded in the hospital mission.

One has to customize its service unit, to provide a dedicated service mix for each different patient group. A dedicated track can be designed to provide grouped access to all the care modalities in an efficient way.

The reimbursement system should prevent price fluctuations and provide hospitals with sufficient financial means for their mission and community service.

Dedicated in-hospital electronic ordering – appointment – planning systems are value enhancing systems to create balance between demand and supply and to allocate demands to the specific imaging modalities and will therefore reduce volumes and duplication.

## Value Delivery and Capture

Service delivery must be aligned to the expectations of patients and referring physicians using patient referral life-cycle management, to reduce no-shows and to improve operations and quality of care.

Gap analysis models can depict perceived services of customers being not aligned with their expectations.

Marketing performance should be measured and be accountable to all stakeholders.

The four dimensions in the strategic balanced scorecard measure the contribution or value of marketing, the strategic position and the overall success of the organization.

## Go to Market

A direct single point of contact is based on personal contacts and regular communications.

Modern times call for modern rules for marketing. Traditional media, while still effective, is slowly dying. With the rise of mobile health technology, marketing becomes mobile and electronic. 72% of internet users said they looked online for health information within the past year [4].

The information should be readable, relevant and tailored to the needs, whereas untargeted content will just be ignored.

Healthcare organizations have options in marketing – from outsourcing to bringing the function completely in-house, and everything in between [5].

## Conclusion

Marketing is all about building exchange relationships to relationships of all the stakeholders. Imaging departments are urged to apply their marketing strategy, implementing a culture of listening to asses the environment, to allign and to establish true long-term commitment. The service chain is strengthened by providing patient-centric and value-based healthcare.

Marketing still has a negative perception in not-for profit organizations, but given the current environmental changes, it is time that decisions makers step up to implement marketing in their hospital organization, to realize its full benefits.
